# Classification of COVID-19 Chest CT Images Based on Ensemble Deep Learning

**DOI:** 10.1155/2021/5528441

**Published:** 2021-04-20

**Authors:** Xiaoshuo Li, Wenjun Tan, Pan Liu, Qinghua Zhou, Jinzhu Yang

**Affiliations:** ^1^Key Laboratory of Intelligent Computing in Medical Image, Ministry of Education, Northeastern University, Shenyang 110189, China; ^2^College of Computer Science and Engineering, Northeastern University, Shenyang 110189, China

## Abstract

Novel coronavirus pneumonia (NCP) has become a global pandemic disease, and computed tomography-based (CT) image analysis and recognition are one of the important tools for clinical diagnosis. In order to assist medical personnel to achieve an efficient and fast diagnosis of patients with new coronavirus pneumonia, this paper proposes an assisted diagnosis algorithm based on ensemble deep learning. The method combines the Stacked Generalization ensemble learning with the VGG16 deep learning to form a cascade classifier, and the information constituting the cascade classifier comes from multiple subsets of the training set, each of which is used to collect deviant information about the generalization behavior of the data set, such that this deviant information fills the cascade classifier. The algorithm was experimentally validated for classifying patients with novel coronavirus pneumonia, patients with common pneumonia (CP), and normal controls, and the algorithm achieved a prediction accuracy of 93.57%, sensitivity of 94.21%, specificity of 93.93%, precision of 89.40%, and F1-score of 91.74% for the three categories. The results show that the method proposed in this paper has good classification performance and can significantly improve the performance of deep neural networks for multicategory prediction tasks.

## 1. Introduction

In March 2020, COVID-19 caused by SARS-CoV-2 has reached global pandemic levels. As of January 2021, the World Health Organization issued a bulletin showing that the cumulative number of confirmed cases worldwide exceeds 91 million, the cumulative number of deaths exceeds 1.9 million deaths, and up to 300,000 new confirmed cases per day, and artificial intelligence methods are one of the important tools for the diagnosis of clinical novel coronavirus pneumonia (COVID-19) [1, 2]. In clinical practice, etiological tests including sputum, pharyngeal swabs, and lower respiratory secretions, such as reverse transcription-polymerase chain reaction (RT-PCR) and gene sequences, are the gold standard for the diagnosis of COVID-19, and nucleic acid testing is widely considered to be the main criterion for discharge after treatment. However, the current epidemiological situation of COVID-19 is dramatically increasing, and a large number of COVID-19 infections are being confirmed every day. Diagnostic methods of nucleic acid testing are faced with long waiting times for test results, certain false negatives, and shortage of testing reagents, and CT image analysis of the chest is also considered an important adjunctive diagnostic tool. For example, almost all patients with COVID-19 have some typical radiological features of chest CT, including ground-glass opacities, multifocal patchy consolidation, and/or interstitial changes in a peripheral distribution [3–8]. Therefore, until definitive results are obtained, rapid differentiation of patient type, based on chest CT images, may be a useful tool to improve diagnosis and to better characterise disease effects.

Deep learning is a part of machine learning whose concept originated from the study of artificial neural networks. Deep learning discovers distributed feature representations of data by combining lower-level features to form more abstract higher-level representations of attribute categories or features. Researchers have made important research progress in previous work by implementing deep learning algorithms to predict and classify pneumonia such as COVID-19 based on chest medical images and other biological indicators. According to Table 1, the existing studies on COVID-19 face the following problems: Wang [10] used a three-dimensional deep neural network to classify COVID-19 and a weakly supervised approach to localize lesion regions by combining the activated regions and unsupervised connected components in the classification network, obtaining 90.1% accuracy and 95.5% ROC. However, this 3D classification model has problems such as long training time. Han [11] designed an attention mechanism-based pooling method applied to 3D data instance prediction that achieved 97.9% accuracy and 99% AUC in COVID-19, CP, and normal classification tasks, but there are problems of limited test set size, for instance, prediction based on 3D data. HORRY [12] proposed a COVID-19 detection method based on migration learning and multimodal image data, but the performance of the generated classification model is insufficient. Pathak [17] proposed a deep bidirectional long-short memory network with a hybrid density network model (DBM) for COVID-19 and non-COVID-19 classification tasks achieving 98.37% accuracy and 98.32% AUC; its dataset size is small with only 1790 sheets. Wang et al. [21] designed an X-ray migration learning and model integration deep learning method for COVID-19, which achieved good results of 96.1% in triclassification prediction tasks, but there are only 140 images of COVID-19 patients in the dataset, and more than 7000 images of common pneumonia patients and normal controls; but this dataset is small and unbalanced.

To address the problems of insufficient accuracy, long training time, and insufficient dataset size in multiclassification prediction tasks [27–29], a COVID-19-assisted diagnosis algorithm based on integrated deep learning is proposed in this paper. The algorithm aims to train deep learning models for small data sets to reduce the training time and the requirements for machine performance. Meanwhile, the integrated learning algorithm integrates multiple deep learning models to improve the performance of multiclassification prediction tasks. The CT image data on this experiment were obtained from a large CT dataset constructed by the China Consortium for Chest CT Image Survey (CC-CCI), including 61,775 CT images from 4,154 patients [1]. Zhang compared them with the assistance of senior radiologists in his study, and the experimental results showed that the AI algorithm showed 92.49% accuracy in many hospitals with practical applications and 98.13% AUROC and also showed good sensitivity in diagnosing COVID-19.

## 2. Materials and Methods

### 2.1. Ensemble Deep Learning Models

**Figure 1:**
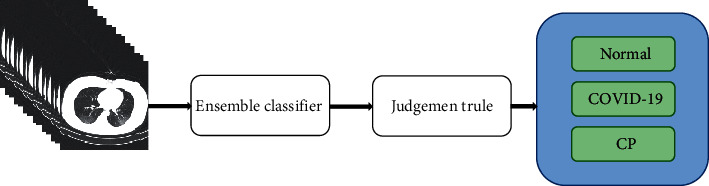
Overall flow of the algorithm. Train multiple deep learning models by dividing subsets, integrate models by stacked idea, and finally output classifier prediction results by setting the threshold probability to 0.5.

For the deep learning model, the prediction performance of the classifier changes when the size of the training set and the number of prediction types of the classifiers change. In Table 2, all classifier types with the corresponding accuracies are shown; the best accuracy is achieved by the classifier M24, which is a binary classifier for classifying COVID-19 and common pneumonia; the worst accuracy is achieved by the classifier M31, which is a triclassifier for classifying COVID-19, common pneumonia, and normal control under subtraining set U1. We can learn that all binary classifiers trained on the subtraining set using the VGG16 deep neural network have better performance, while the multiclassifiers generated on the subtraining set have poor prediction performance. Therefore, our method integrates a triple classifier and five binary classifiers to form a cascaded classifier. Finally, by analyzing the performance differences among the classifiers, we reduce the deviation before the predicted and true values by the stacked idea and thus output the prediction results to improve the classification performance. The overall flow chart is shown in Figure 1.

In order to improve the performance of the classification model trained by deep neural network for the recognition of three different types of patients such as new coronavirus pneumonia, our algorithm proposes to combine the stacked algorithm with the VGG16 deep learning pretraining model. Firstly, the training set is partitioned into several disjoint subtraining sets, and several binary classifiers and a triple classifier based on the VGG16 model are trained on different subtraining sets; secondly, all classifiers are integrated by stacked idea in ensemble learning to form a cascade classifier; and finally, the prediction results are output according to the cascade classifier. In this paper, the training set is divided into six subtraining sets and a triclassifier, and five binary classifiers are trained, where the discriminant type of each classifier is shown in Table 2.

### 2.2. VGG (Visual Geometry Group)

VGG is a classic deep convolution neural network jointly developed by Oxford University's Visual Geometry Group and Google DeepMind researchers [30]. The network is a related research work on Large Scale Visual Recognition Challenge 2014. Its main work is to prove that increasing the depth of the network can affect the final performance of the network to a certain extent. VGG has two main structures: VGG16 and VGG19. An important improvement on VGG16 is that several continuous 3 × 3 convolution kernels replace the larger convolution kernels in AlexNet. For a given receptive field, it is better to use an accumulated small convolution kernel than a large convolution kernel because a multilayer nonlinear layer can increase the depth of the network to ensure learning more complex patterns, and the cost is relatively small.

### 2.3. Transfer Learning

Transfer learning means to transfer or extend the representations learned by the CNN in previous tasks to new tasks or new fields [31]. This paper is based on the VGG16 deep neural network, and the VGG16 model has been pretrained on large tagged natural image data sets such as ImageNet, so that the train time and the amount of calculation can be significantly reduced. At the same time, in order to better extract features, the size of the training and test images input to the VGG16 model in this experiment is not adjusted to the model preset 224 *∗* 224, but the size of the original chest CT image is maintained, which is 512 *∗* 512.

### 2.4. Stacked Generalization

Stacked generalization is an important ensemble learning idea proposed by David H. Wolpert in 1992 [32]. Stacking generalization refers to the scheme of providing information from one group of classifiers to another group of classifiers before forming the final prediction result. The prominent feature of stacking generalization is that the information constituting the classifier network comes from multiple subsets of the training set, and the original training set is divided into multiple subsets of training sets. Each subtraining set is used to collect bias information about the generalization behavior of the data set so that this bias information fills the classifier network. Stacking generalization is a method to estimate and correct the deviation from the constituent classifier to the training set provided.

### 2.5. Training Set Partition

Based on the idea of stacked generalization in ensemble learning, the total set *U* is divided into *m* subsets, *U*_*i*_ are the subsets, and intersection of all subsets is null; the number of subsets *U*_*i*_ is the number of total set *U* divided by m:

(1) $$\begin{array}{c} U=U_{1}\cup U_{2}\cup U_{3}\cdots U_{m},\\ \phi =U_{1}\cap U_{2}\cap U_{3}\cdots U_{m},\\ \frac{N}{m}=\text{Num}\left(U_{i}\right),\;i=1,2,3,\ldots ,m. \end{array}$$

## 3. Results and Discussion

### 3.1. Dataset

As shown in Table 3, CT images of 1417 patients with NCP, common pneumonia, and normal controls were used to train and test the prediction model proposed in this paper. The prediction model was trained with 14,400 images of 328 patients [1], including 128 patients with common pneumonia, 115 patients with NCP, and 85 normal controls. Performance tests were conducted using 139,852 slices of 1,089 patients, including 76,000 slices from 675 NCP patients, 18,852 slices from 256 patients with common pneumonia, and 45,000 slices from 158 normal controls to test the improvement of the performance of the triclassifiers trained by the deep learning algorithm.

### 3.2. Evaluation Measures

This method used accuracy, precision, recall (or sensitivity), *F*1-score, and specificity to measure and analyze the performance of the ensemble learning model. Accuracy is the classifier's ability to correctly predict all samples, and precision is the classifier's ability not to predict negative samples as positive. Recall is the classifier's ability to classify all those with the disease correctly (true positive rate). *F*1-score is the weighted average of precision and recall. Specificity is the ability of the classifier to correctly identify patients without the disease (true negative rate). TP is true positives, TN is true negatives, FP is false positives, and FN is false negatives. The formulas of the measures are given below:

(2) $$\begin{array}{c} \text{accuracy}=\frac{\text{TP}+\text{FN}}{\text{TP}+\text{TN}+\text{FN}+\text{FP}},\\ \text{precision}=\frac{\text{TP}}{\text{TP}+\text{FP}},\\ \text{sensitivity}=\text{recall}=\frac{\text{TP}}{\text{TP}+\text{TN}},\\ F1-\text{score}=2*\frac{\text{sensitivity}*\text{precision}}{\text{sensitivity}+\text{precision}},\\ \text{specificity}=\frac{\text{TN}}{\text{TN}+\text{FP}}. \end{array}$$

### 3.3. Results and Discussion

**Figure 2:**
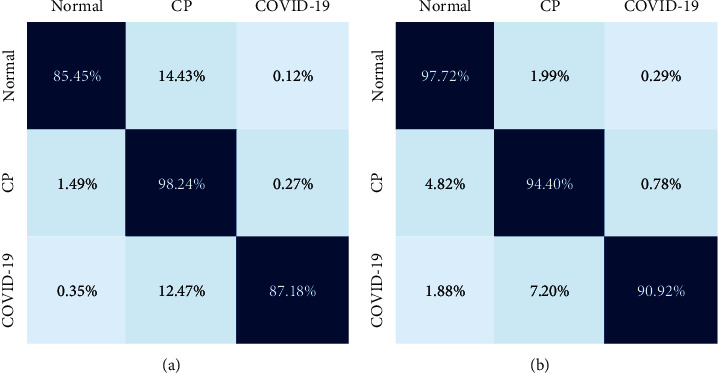
Compare the accuracy, sensitivity, precision, F1-score, and specificity under deep learning based on VGG16 and based on the combination of ensemble learning and VGG16.

This method trains and tests a triclassifier based on VGG16 deep neural network only and a triclassifier based on VGG16 deep neural network with integrated learning under the same training set conditions, respectively. The performance of each method is compared by accuracy, specificity, and sensitivity on the test dataset. According to Figure 2, it can be seen that even the triclassifier trained solely based on the VGG16 model showed good results in identifying neocoronary pneumonia, common pneumonia, and normal controls, while the performance of the cascade model combining the deep neural network and the integrated learning algorithm was significantly improved.

**Figure 3:**
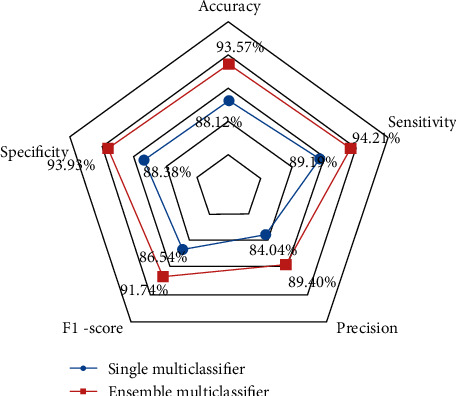
Results of COVID-19, CP, and normal evaluated under two methods. (a) Accuracy evaluation of a triclassifier model based on VGG16. (b) Accuracy evaluation of a triclassifier model based on a combination of integrated learning and VGG16.

The experimental results in this paper show that the cascade classifier constructed by combining the deep learning algorithm with the integrated learning algorithm can significantly improve the multiclassification prediction accuracy of the model. According to Figure 3, it can be seen that among the prediction results using only the triclassifier M31, the normal control group is predicted as common pneumonia and new coronary pneumonia is predicted as common pneumonia, and these two false predictions occur more frequently, with the false prediction rates of 14.43% and 12.47%, respectively. For these two classification cases with high error rates, two classifiers M24 and M25 with prediction performance over 95% were trained in this paper by the subtraining sets U5 and U6 for improving the predictions with high error rates, and the number of these two errors was significantly reduced to 1.99% and 7.20%, respectively, thus greatly improving the prediction accuracy of the normal control group and new coronary pneumonia, especially new coronary pneumonia identified as normal pneumonia was greatly reduced in the error rate. In the cascade classifier, the number of discrimination errors increases when the original discrimination errors are less, such as judging normal control as new coronary pneumonia and judging new coronary pneumonia as the normal control group. As can be seen from Figure 2, the incorrect discrimination error rate of judging normal pneumonia as neocoronary pneumonia only increased from 0.27% to 0.78%, and there was no significant increase in the incorrect discrimination rate.

According to Figure 3, the accuracy, specificity, sensitivity, precision, and F1-score of the integrated model based on the combination of VGG16 deep neural network and integrated learning algorithm are 93.57%, 93.93%, 94.21%, 89.40%, and 91.74%, respectively, while the accuracy, specificity, sensitivity, precision, and F1-score of the single triclassifier trained based on VGG16 algorithm are 88.12%, 88.38%, 89.19%, 84.04%, and 86.54%, respectively. Compared with the single multiclassifier, the accuracy increased by 5.45%, the specificity increased by 5.55%, the sensitivity increased by 5.02%, the precision increased by 5.36%, and the F1-score increased by 5.2%; all the indicators were significantly improved.

## 4. Conclusions

In this paper, we propose an algorithm based on the combination of VGG16 deep neural network and ensemble learning with the aim of improving the performance of deep neural networks for multiclassification prediction tasks. The experimental results show that the VGG16 deep neural network combined with the integrated learning approach can significantly improve the classification performance compared with the VGG16 deep neural network-based integrated learning algorithm under the same conditions, which plays an important role in the rapid identification of patients with novel coronavirus pneumonia. The method proposed in this paper has the following drawbacks: (1) the training and testing of the classification model is only utilized on 2D images, while the rich spatial information preserved in the 3D structure is not utilized; (2) only the VGG model is used and some new network techniques are not tried; and (3) the publicly available dataset used in this method is not the original DICOM data format, and the image is lost in the process of data format conversion pixel information.

## Figures and Tables

**Table 1:** Overview of methods and quantitative results toward COVID-19 classification.

Author	Dataset	No. of images	Method	Quantitative results indicators
Gao [9]	Internal	791	XGBoost	Acc = 94.34%; Sens = 83.33%
Wang [10]	Internal	540	3D CNN	Acc = 90.1%;ROC = 95.5%
Han [11]	Internal	460	Attention mechanism + 3D multiple instance learning	Acc = 97.9%; AUC = 99.0%
J. HORRY [12]	COVID-CT dataset	746	VGG19	Acc = 84%
A. Waheed [13]	COVID-19 chest X-ray dataset [14–16]	932	VGG16 + ACGAN	Acc = 95%; Sens = 90%
Pathak [17]	Chest CT images [18]	1790	DBM	Acc = 98.37%; AUC = 98.32%
Y. Oh [19]	JSRT [20]	502	ResNet-18	Acc = 88.9%; Spec = 96.4
Wang [21]	RSNA [22]; chest X-ray [23]	18567	ResNet-101 + ResNet-102	Acc = 96.1%
Ouyang [24]	Internal	2796	Attention mechanism + 3D CNN	Acc = 87.5%; AUC = 94.4%; Sens = 86.9%
T. Siswantining [25]	Internal	170	CNN + SVM + NN	Acc = 95%
Dong [26]	Internal	640	DCNN	Acc = 93.64 ± 1.42% Sens = 93.28 ± 1.5% Spec = 94.0 ± 1.56%
Zhang [1]	CC-CCI [1]	61775	3D Resnet-18	Acc = 92.49%; Sens = 94.93%; Spec = 91.13%

Internal is the nonpublic dataset.

**Table 2:** Functions and accuracy of all classifiers.

Classifier name	Classifier type	Discriminate type	Training set	Accuracy (%)
M3	Multiclassifier	[COVID-19, CP, normal]	U	88.12
M31	Multiclassifier	[COVID-19, CP, normal]	U1	85.57
M21	Binary classifier	[COVID-19, (CP, normal)]	U2	95.69
M22	Binary classifier	[CP, (COVID-19, normal)]	U3	94.07
M23	Binary classifier	[Normal, (COVID-19,CP)]	U4	95.91
M24	Binary classifier	[COVID-19, CP]	U5	96.49
M25	Binary classifier	[CP, normal]	U6	95.73

**Table 3:** Introduction to dataset size.

Cohort	COVID-19			Common pneumonia			Normal		
	Patients	Scans	Slices	Patients	Scans	Slices	Patients	Scans	Slices
Train	115	183	4800	128	303	4800	85	108	4800
Validate	115	183	480	128	303	480	85	108	480
Test	675	1180	76000	256	441	18852	158	364	45000
Total	790	1363	80800	384	744	23652	243	472	49800

## Data Availability

The data used to support the findings of this study are available at http://ncov-ai.big.ac.cn/download?lang=en.
